# Antibacterial efficacy of berry juices against *Bacillus cereus* relative to their phytochemical composition and antioxidant properties

**DOI:** 10.1038/s41598-024-79155-y

**Published:** 2024-11-16

**Authors:** Kamil Królak, Sylwia Ścieszka, Edyta Kordialik-Bogacka, Joanna Oracz, Maciej Ditrych, Tomasz Szczygieł, Katarzyna Dybka-Stępień, Anna Otlewska, Dorota Żyżelewicz

**Affiliations:** 1 Institute of Fermentation Technology and Microbiology, Faculty of Biotechnology and Food Sciences, Lodz University of Technology, 171/173 Wólczańska, 90-530 Lodz, Poland; 2 Institute of Food Technology and Analysis, Faculty of Biotechnology and Food Sciences, Lodz University of Technology, 2/22 Bohdana Stefanowskiego, 90-537 Lodz, Poland

**Keywords:** *Bacillus cereus*, Berry juices, Antibacterial, Antioxidant, Anthocyanins, Organic acids, Biochemistry, Biotechnology, Microbiology

## Abstract

Ensuring the safety and stability of minimally processed foods using natural preservatives is of great scientific and commercial interest in modern biotechnology. Berry juice supplementation is increasingly recognized within this field. This study investigated the effectiveness of juices from four berry species *Aronia melanocarpa*, *Ribes nigrum*, *Vaccinium macrocarpon*, and *Sambucus nigra*, against the food pathogen *Bacillus cereus*. Overall, the antibacterial potency of juice supplements (up to 10% *v/v* in tryptic soy broth) followed the order of chokeberry > blackcurrant > cranberry > elderberry, with the latter showing no inhibitory effects. Notably, chokeberry and elderberry juices presented lower acidity and significantly greater phenolic contents (*p* < 0.05) than blackcurrant and cranberry juices did, suggesting that *B. cereus* susceptibility is not strictly dependent upon low extracellular pH or elevated anthocyanin levels. Instead, it is inferred to correlate with pro-oxidative effects induced directly at the intracellular level. Accordingly, this paper discusses the antioxidative, acidic, and lipophilic attributes of juices and their constituent fractions, including anthocyanins, to elucidate their biopreservative potential. The results of this study increase our understanding of the antibacterial susceptibility of *B. cereus*.

## Introduction

Traditionally, microbial food preservation relies heavily on thermal techniques such as pasteurization, sterilization, hot infusion, or steaming, which operate at temperatures between 60 °C and 150 °C^1^. However, in recent years, there has been increasing adoption of milder heat preservation methods across various sectors of the food and beverage industry^2,3^. These methods aim to save energy, promote environmental sustainability, and may even result in reduced nutritional and sensory degradation^4^. Furthermore, there has been a noticeable shift in physicochemical preservation practices toward embracing the “clean” label concept, prioritizing the reduction of synthetic additives or their substitution with natural antioxidant compositions, such as fruit juices, which are also more acceptable by consumers from a sensory perspective.

Despite their benefits, these modern preservation methods present challenges to the microbiological quality and safety of many foods, particularly those of plant origin. Mild or ambient temperature food processing and/or supplementation with fruits may unintentionally trigger the germination of spores or the growth of vegetative *Bacillus cereus*, a gram-positive, facultative anaerobic, spore-forming bacterium naturally found in various types of soil and, consequently, in certain plant-based raw materials. The consequences of its growth may not only affect food flavor and structure due to biofilm formation and the release of hydrolytic extracellular enzymes^5^ but also raise health concerns associated with cereulide formation, an emetic toxin that can develop during food processing and storage^6^.

Accordingly, there is growing scientific and commercial interest in the development of effective natural preservative agents, i.e., biopreservatives, for preventing or retarding biological and chemical deterioration in minimally processed foods^7,8^. Fruits indeed hold significant promise for biopreservative applications, as they represent a virtually inexhaustible source of natural antimicrobials and antioxidants. Berries, in particular, are recognized for their high content of weak organic acids and/or phenolic compounds, which have demonstrated efficacy against various human pathogens, including *Bacillus cereus*^9–13^. Importantly, our recent studies on the microbial stability of natural tea beverages revealed that juice supplements from phenolic-rich and high-antioxidant elderberry (*Sambucus nigra*) and chokeberry (*Aronia melanocarpa*) fruits, as documented in the literature^14–17^, differ in their ability to mitigate *B. cereus* contamination, one of the major bacterial threats identified in association with a recently embraced cold-infusion process^18^. This finding highlights the critical role of the specific composition of berry-derived additives in shaping biopreservation outcomes. Consequently, we sought to investigate the biochemical factors contributing to the differences in antibacterial efficacy against *B. cereus* among these berry juices, a topic not comprehensively addressed in the literature. This information could serve as the foundation for the optimal utilization of specific berry products in tea technology and other food and beverage applications aimed at controlling *B. cereus* contamination.

Exploring the relationships among the phytochemical composition, antioxidant capacity and antibacterial activity of individual berry juices as well as their fractional components is expected to provide general insights into the susceptibility of *B. cereus*, thereby revealing the nature of growth-inhibitory and nonantagonistic compounds.

## Materials and methods

### Chemical reagents

Sodium hydroxide (NaOH), ethyl acetate, ethanol (96%), and methanol were purchased from Avantor Performance Materials Poland S.A. (Gliwice, Poland). DPPH (1,1-diphenyl-2-picrylhydrazyl), ABTS (2,2′-Azino-bis(3-.

ethylbenzothiazoline-6-sulfonic acid) diammonium salt, potassium persulfate, Trolox (6-hydroxy- 2,5,7,8-.

tetramethylchroman-2-carboxylic acid), Folin-Ciocalteu reagent, sodium carbonate, gallic acid, catalase from bovine liver, and phenolic and organic acid standards were purchased from Sigma‒Aldrich (Merck, Darmstadt, Germany). Acetonitrile for HPLC was purchased from Honeywell (USA), whereas phenolphthalein and hydrochloric acid (35–38%) were purchased from Chempur (Piekary Śląskie, Poland).

### Berry juices

Four types of commercially available NFC (not from concentrate) berry juices — elderberry (*Sambucus nigra*), chokeberry (*Aronia melanocarpa*), blackcurrant (*Ribes nigrum*), and cranberry (*Vaccinium macrocarpon*) — were sourced from Batom (Kraków, Poland) for this study. Juices from three distinct production batches were used to ensure variability and representativeness in the analysis. According to the manufacturer’s declaration, the juices were cold-pressed from whole fruits and subjected to mild continuous flow pasteurization, ensuring that the temperature did not exceed 85 °C, with no additional preservation techniques or agents applied. To confirm that the juices would not introduce additional microorganisms, a microbial analysis was conducted. Overall, the results demonstrated the absence of mesophilic, psychrophilic, *Enterobacteriaceae*, and lactic- and spore-forming bacteria in the juice microflora, ruling out juices as potential sources of contamination.

### SPE fractionation of berry juices

Two milliliters of a berry juice sample were partitioned into two fractions, namely, phenolic and anthocyanin fractions, via solid-phase extraction (SPE) with C-18 columns, following the procedure outlined by^19^. After drying and reconstitution, qualitative and quantitative analyses were conducted on the fractions as detailed in section *UHPLC-DAD and UHPLC-ESI-HRMS/MS analysis of phenolic compounds*. Prior to the antibacterial assessments, the dried fractions were redissolved in tryptic soy broth (TSB medium).

### Survival of *B. cereus* in the presence of berry juices and their individual components

The study utilized two bacterial strains: *Bacillus cereus* AK1, a newly isolated strain from cold-brewed green tea (accession number OQ875857 in the GenBank National Centre for Biotechnology Information database), and *Bacillus cereus* ŁOCK 0807, obtained from the Pure Culture of Industrial Microorganisms of the Institute of Fermentation Technology and Microbiology ŁOCK 105 (Łódź, Poland). Prior to the experiments, the strains stored in cryovials at -80 °C were activated through two successive subcultures in TSB medium (Merck, Darmstadt, Germany; pH 7.4 ± 0.1). Bacterial growth was monitored in media supplemented with a single type of juice at final concentrations of 5%, 7.5%, or 10% *(v/v)*, either in its native (acidic) or neutralized form. These concentrations mimic typical commercial ready-to-drink beverages. In this context, higher supplementation levels had been found to be detrimental from a sensory perspective. For the neutralized juices, the pH was adjusted to 7.4 (± 0.1) using a 1 M NaOH solution. With respect to the fractional components of individual berry juices —phenolics and anthocyanins — the amounts used in the experiments were 3 and 5 milligrams per milliliter of the sample solution, respectively. The prepared solutions were inoculated with a 1% (*v/v*) 24-hour liquid inoculum containing approximately 10^6^ bacterial colony-forming units per milliliter (CFU/mL) and then incubated at 30 °C for 24 h. For comparison purposes, positive controls were inoculated with TSB without juices or their fractions. The viable counts of individual *B. cereus* strains were evaluated at specific time intervals during the incubation period via the plate method. This included measurements at 0, 1, 6, 10, and 24 h for the juice supplements and at 0 and 24 h for the fractional components. The toxicity of growth-inhibitory juice supplements toward *B. cereus* cells was also assessed after 24 h of treatment in the absence and presence of 500–10,000 U of catalase. After each incubation period, the cultures were diluted and spread on tryptic soy agar (TSA) plates and then incubated for 24 h at 30 °C. The viable counts were expressed in CFU/mL and alternatively calculated as a percentage of the control.

### pH and acidity of the juices

The pH of the examined juices was determined via a pH meter CP-411 (Elmetron, Zabrze, Poland). The titrable acidity (TA) analysis was performed using a 0.1 M solution of sodium hydroxide and a 1% solution of phenolphthalein. The TTA results were expressed as the volume (mL) of 0.1 M NaOH required to titrate one milliliter of a juice sample (mL 0.1 M NaOH/mL).

### Antioxidant capacity of the juices

The antioxidant capacity (AC) of the juices was determined via the DPPH and ABTS methods, following the protocols described by^20,21^. The reduction of DPPH• radicals was measured at a wavelength of 517 nm, while the reduction of ABTS•+ radicals was measured at a wavelength of 734 nm via a Multiskan SkyHigh Microplate Spectrophotometer (Thermo Fisher Scientific, Waltham, MA, USA). The DPPH and ABTS antiradical activities of the juices were calculated using standard curves with Trolox as the reference compound and expressed as milligrams of Trolox equivalents per liter of the juice sample (mg TE/L).

### Total phenolic content of the juices via spectrophotometry

The total phenolic content (TPc_**F−C**_) of the berry juices via spectrophotometry was determined following the Folin‒Ciocalteu (F‒C) method as described by^22^. The analysis was conducted via a Multiskan SkyHigh Microplate Spectrophotometer (Thermo Fisher Scientific, Waltham, MA, USA) and 96-well plates. The TPc_**F−C**_ in the samples was calculated using a standard curve for gallic acid and expressed as milligrams of gallic acid equivalents per liter of the juice sample (mg GAE/L).

### UHPLC-DAD and UHPLC-ESI-HRMS/MS analysis of phenolic compounds

UHPLC-DAD and UHPLC-ESI-HRMS/MS analyses of phenolic compounds in the juice samples were performed via a UHPLC + Dionex UltiMate 3000 liquid chromatography system (Thermo Fisher Scientific Inc., Waltham, MA, USA) equipped with a diode array detector and a Transcend™ TLX-2 multiplexed LC system coupled to a Q-Exactive hybrid quadrupole‒orbitrap mass spectrometer with a heated electrospray ionization (HESI-II) source (Thermo Scientific, Hudson, NH, USA) according to^23^, with some modifications. Juice samples were diluted in mobile phase at a ratio of 1:10 (*v/v*) and filtered through a nylon membrane filter (0.22 μm pore size) prior to analysis. The chromatograms were recorded at 270 nm for flavan-3-ols and hydroxybenzoic acids, at 320 nm for hydroxycinnamic acids, and at 520 nm for anthocyanins. The identification of phenolic compounds was performed by matching their retention times, spectral characteristics, full-scan mass spectra in negative and positive ionization modes, and MS/MS fragmentation patterns with those of pure standards analyzed under identical conditions. The quantification of individual phenolic compounds was carried out using the external standard method. The results were expressed as milligrams of phenolic compounds per hundred milliliters of the juice sample (mg/100 mL). The concentration ranges, correlation coefficients (R^2^), limits of detection (LODs), and limits of quantification (LOQs) for the phenolic compounds investigated are given in Table S1.

### HPLC‒DAD analysis of organic acids

HPLC-DAD analysis of organic acids in the juice samples was performed via a UHPLC + Dionex UltiMate 3000 liquid chromatography system (Thermo Fisher Scientific Inc., Waltham, MA, USA) equipped with a diode array detector according to^24^, with some modifications. Juice samples were diluted in mobile phase at a ratio of 1:10 (*v/v*) and filtered through a nylon membrane filter (0.45 μm pore size) prior to analysis. Chromatographic separation of the phenolic compounds was achieved using a A11606 C18 column (2.1 × 150 mm, particle size 2.6 μm; ATC, Waltham, MA, USA). The column was maintained at 30 °C, and the flow rate was 0.8 mL/min. The mobile phase solutions were 20 mM NaH_2_PO_4_ buffer, pH 2.3 (A), and acetonitrile (B). The following gradient program was used: 0 min, 0% B; 0–5.5 min, from 0 to 80% B; 5.5–10.5 min, constant at 80% B; 10.5–19.5 min, from 80 to 0% B; 19.5–25 min, constant at 0% B. Chromatograms were recorded at 210 nm. The identification of organic acids was based on a comparison of the retention times of analytes with those of available standards (oxalic acid, tartaric acid, malic acid, ascorbic acid – AsA, citric acid – CA, and fumaric acid). The external standard method was used to determine the concentrations of individual organic acids. The results were expressed as milligrams of organic acids per hundred milliliters of the juice sample (mg/100 mL).

### Reproducibility

All assays were performed on three independently produced replicates of each berry juice, and the data are expressed as means ± standard deviations. Figures were created via GraphPad Prism (version 9.00, GraphPad Software Inc., San Diego, CA) and Excel (Microsoft 365, USA). Statistical analyses were performed via R software (version 4.2.2; PBC, Boston, MA). The normality of the distribution was assessed with the Shapiro‒Wilk test, and variance homogeneity was evaluated using Bartlett’s test. Differences among the examined parameters were analyzed using one-way ANOVA, followed by Tukey’s honest significance test (HSD) and Duncan’s test (MRT), with a significance level of α = 0.05. The Kruskal‒Wallis test was used to evaluate differences between juice-treated and untreated bacterial cells (assessments involving catalase and fractional components of the juices).

## Results

### Effects of juice supplements on *B. cereus*

**Fig. 1:**
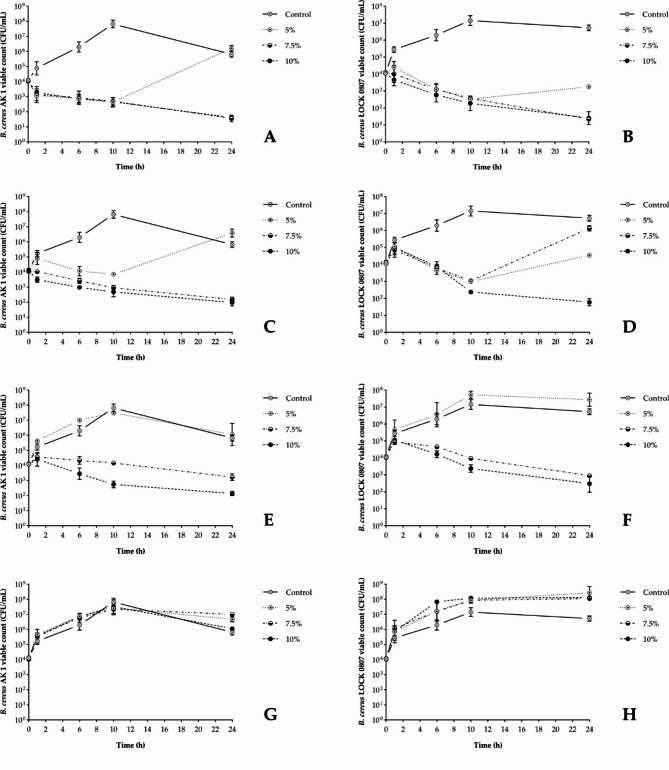
Effects of the analyzed berry juices and their concentration (5; 7.5; 10% *v/v*) on the survival of *B. cereus* strains (AK1 – on the left; ŁOCK 0807 – on the right). (**A**, **B**) Chokeberry juice (5% *v/v*, pH 6.75; 7.5% *v/v*, pH 6.55; 10% *v/v*, pH 6.25); (**C**, **D**) Blackcurrant juice (5% *v/v*, pH 6.05; 7.5% *v/v*, pH 5.30; 10% *v/v*, pH 4.95); (**E**, **F**) Cranberry juice (5% *v/v*, pH 6.27; 7.5% *v/v*, pH 5.87; 10% *v/v*, pH 5.17); (**G**, **H**) Elderberry juice (5% *v/v*, pH 6.70; 7.5% *v/v*, pH 6.40; 10% *v/v*, pH 6.10).

To mimic the application of juices in the food and beverage industry, the growth medium (TSB) was supplemented with a final juice concentration of 5%, 7.5%, or 10% *(v/v)*, whereas the control samples were left without supplementation. Figure 1 shows the growth pattern of *B. cereus* in the nonstressed control culture, among others, with an initial viable count of approximately 10^4^ CFU/mL. After 10 h of incubation, the viable count of the examined strains increased by a maximum of 3–4 log CFU/mL, reaching a minimum of 10^5^ viable cells per milliliter after 24 h. When exposed to berry juice supplements, both strains displayed roughly similar viable count responses; however, the reference strain demonstrated slightly greater resilience to the applied treatments.

Importantly, elderberry juice had no inhibitory effects on the tested strains, regardless of the concentration used in the study. In fact, this kind of supplementation even promoted the growth of *B. cereus*, increasing bacterial enumeration by more than one order of magnitude after 24 h of incubation compared with that of the control samples (Fig. 1G, H). Furthermore, no growth inhibition was observed with the 5% (*v/v*) cranberry juice supplement (pH 6.27), and for the chokeberry and blackcurrant juices applied at this concentration (pH 6.75 and 6.05, respectively) bacterial cell outgrowth was noted after 24 h (Fig. 1C-F). Neither of the cranberry juice supplements caused a reduction in the viable counts within the first 60 min of exposure, relative to the initial inocula of both tested strains, a pattern also observed for the blackcurrant juice supplements and the ŁOCK 0807 strain. Moreover, the growth of the reference strain resumed after 10 h in the presence of 7.5% (*v/v*) blackcurrant juice (pH 5.30), whereas viable counts for both strains remained relatively constant for the 7.5% (*v/v*) cranberry juice supplement (pH 5.87). Finally, a decrease of at least 1 log CFU/mL in viable count was observed after 24 h in the presence of 10% (*v/v*) chokeberry (pH 6.25), blackcurrant (pH 4.95), or cranberry (pH 5.17) juice compared with the initial inoculum concentration of approximately 10^4^ CFU/mL. An equally strong inhibitory effect was noted for the 7.5% (*v/v*) chokeberry juice supplement (pH 6.55). Notably, this agent was the only agent capable of reducing and maintaining the viable counts of both tested strains below the initial inoculum level throughout the entire incubation period, ultimately reducing the number of viable cells to less than 100 per mL.

### Effects of neutralized juices on *B. cereus*

Additional experiments were conducted to evaluate the antibacterial activity of berry juices under neutral pH conditions. Analogously to the previous test, the neutralized juices were added to TSB medium (pH 7.4) at final concentrations of 5%, 7.5%, or 10% *(v/v)*.

Upon neutralization, blackcurrant, cranberry, and elderberry juices presented no evident inhibition of.

**Fig. 2:**
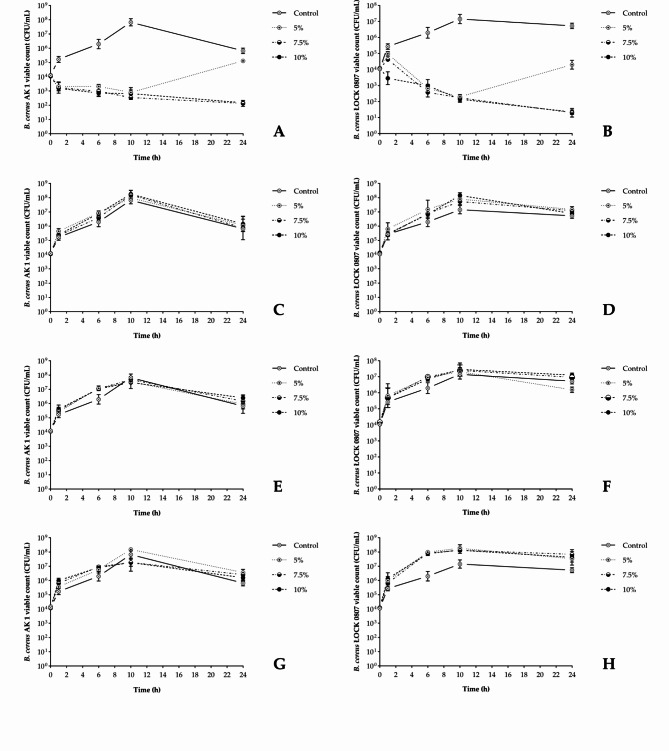
Effects of the neutralized berry juices (pH 7,40 ± 0,10) and their concentration (5; 7.5; 10% *v/v*) on the survival of *B. cereus* strains (AK1 – on the left; ŁOCK 0807 – on the right). (**A**, **B**) Chokeberry juice; (**C**, **D**) Blackcurrant juice; (**E**, **F**) Cranberry juice; (**G**, **H**) Elderberry juice.

*B. cereus* growth compared with the control samples, regardless of the final concentration applied (Fig. 2C-H). Moreover, the supplements seemed to even provide support for bacterial growth. In contrast, the neutralized chokeberry juice supplements yielded nearly identical growth patterns in both tested strains (Fig. 2A, B) as was observed in the native juice-stressed cultures (Fig. 1A, B).

### Effects of juice fractional components on *B. cereus*

**Fig. 3:**
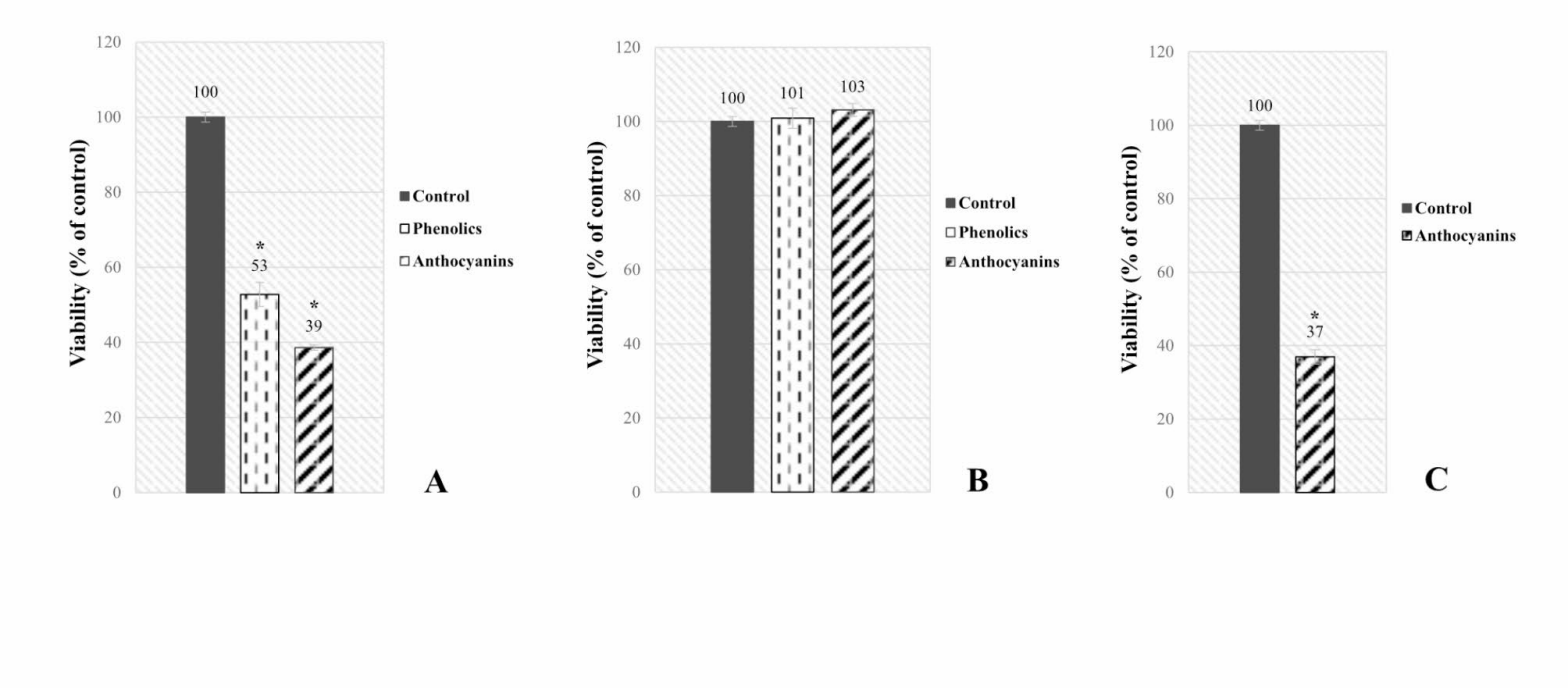
Effects of the fractional components of the juices – phenolics and anthocyanins – on the viability of *B. cereus* AK1 after 24 h of incubation in TSB medium. (**A**) Chokeberry juice (*anthocyanins*: 70% cyanidin-3-*O*-galactoside, 15% cyanidin-3-*O*-arabinoside, 14% cyanidin-3-*O*-glucoside, 1% co-eluted flavonols; *phenolics*: 36% neochlorogenic acid, 28% chlorogenic acid, 12% quercetin 3-*O*-rutinoside, 11% quercetin 3-*O*-glucoside, 9% protocatechuic acid, 4% quercetin 3,7-di-*O*-glucoside); (**B**) Elderberry juice (*anthocyanins*: 79% cyanidin-3-*O*-sambubioside, 8% cyanidin-3-*O*-sambubioside-5-O-glucoside, 13% co-eluted flavonols; *phenolics*: 48% quercetin 3-*O*-glucoside, 44% chlorogenic acid, 4% quercetin 3-*O*-rutinoside, 4% quercetin 3,7-di-O-glucoside); (**C**) Blackcurrant juice (*anthocyanins*: 70% cyanidin-3-*O*-rutinoside, 10% cyanidin-3-*O*-glucoside, 7% delphinidin-3-*O*-rutinoside, 1% delphinidin-3-*O*-glucoside, 12% co-eluted flavonols). * *p* ≤ 0.05, Kruskal‒Wallis test against non-treated control.

To elucidate the growth-inhibitory effects of individual berry juices on *B. cereus*, chromatographic and antibacterial assessments were also conducted on their fractional components, namely, phenolics and anthocyanins. Figure 3 provides insight into the antibacterial activity as well as the qualitative and quantitative composition (% *m/m*) of each fraction investigated in the study. Due to the nonexistent yields of the anthocyanin and phenolic fractions from cranberry juice, as well as the phenolic fraction from blackcurrant juice obtained via SPE, no assessments could be conducted, and therefore no results are presented for these fractions. This finding indicates the trace content of phenolic compounds, including anthocyanins, in cranberry and blackcurrant juices, as further detailed in Table 1.

The findings demonstrate that anthocyanins obtained from chokeberry (1.6 µg/mL, data not shown – d.n.s.) and blackcurrant juices (0.3 µg/mL, d.n.s.) exhibited significant antibacterial effects, reducing *B. cereus* viable counts by 61–63% relative to those of the control culture. However, the phenolic fraction of chokeberry juice (0.4 µg/mL, d.n.s) displayed noticeably weaker activity. Interestingly, neither supplementation with anthocyanins (3.9 µg/mL, d.n.s.) nor phenolics (0.5 µg/mL, d.n.s.) from elderberry juice affected the viable bacterial count compared with the control after 24 h of incubation. Importantly, the HPLC analysis revealed significant variations in the chemical composition of the anthocyanin fractions among all three berry species with cyanidin-3-*O*-galactoside, cyanidin-3-*O*-sambubioside, and cyanidin-3-*O*-rutinoside accounting for 70–80% of the total “anthocyanin” content of the chokeberry, elderberry, and blackcurrant juices, respectively. These findings align well with the literature^14,16,25,26^. With respect to the phenolic fractions, minimal differences were detected between chokeberry juice and elderberry juice, except for the additional presence of neochlorogenic acid and protocatechuic acid in the former.

### Effects of 10% (*v/v*) juice supplementation on *B. cereus* coadministered with catalase

**Fig. 4:**
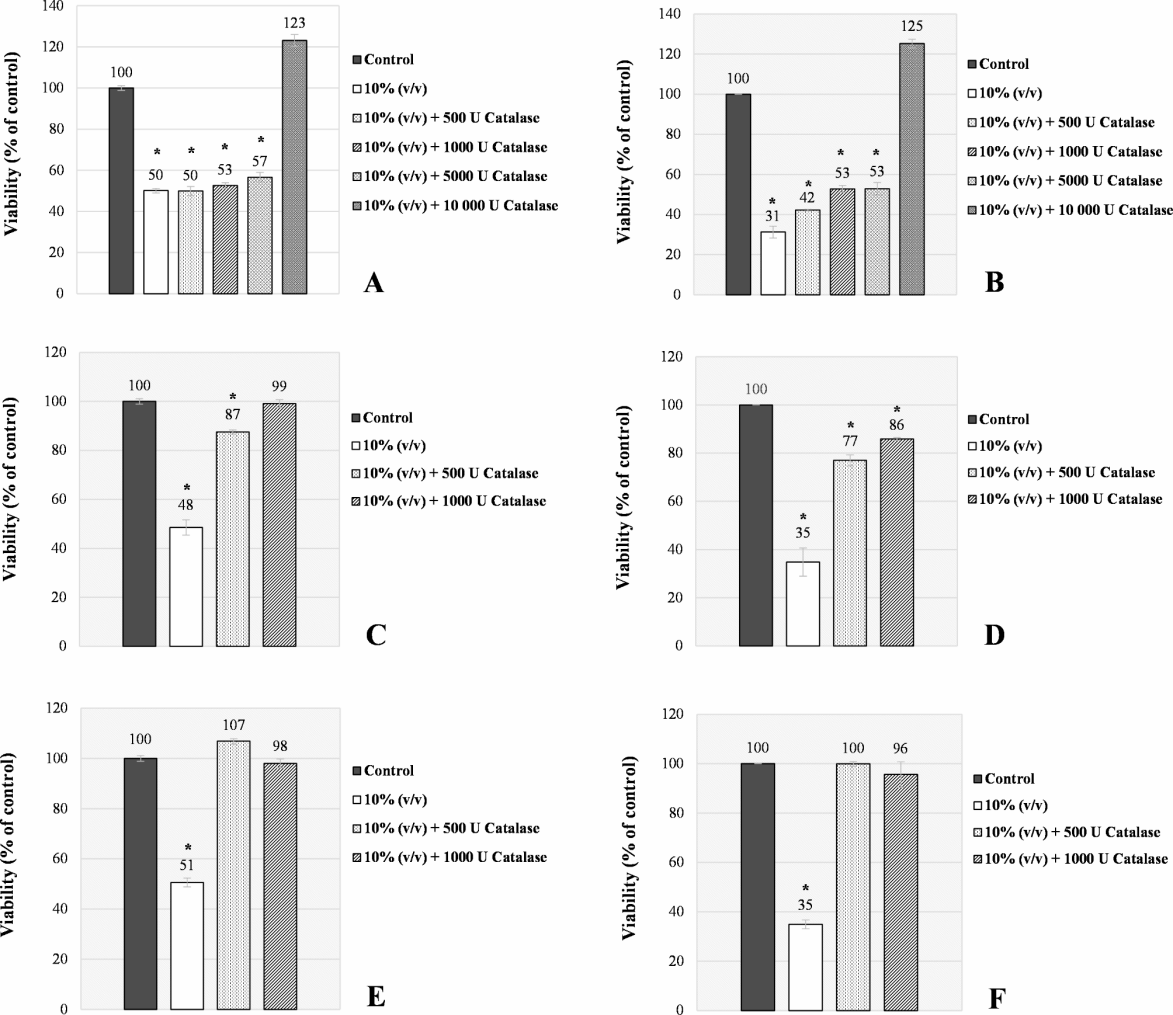
Toxicity of berry juices toward *B. cereus* cells (AK1 – on the left; ŁOCK 0807 – on the right) after 24 h treatment in the absence and presence of 500 − 10,000 U of catalase. (**A**, **B**) Chokeberry juice; (**C**, **D**) Blackcurrant juice; (**E**, **F**) Cranberry juice. * *p* ≤ 0.05, Kruskal‒Wallis test against non-treated control.

To investigate the potential mechanisms underlying the inhibitory effects of chokeberry, blackcurrant, and cranberry juices, antibacterial assessments were also conducted with catalase, a hydrogen peroxide–decomposing enzyme (Fig. 4).

Depending on the dosage applied, treating the juice-stressed *B. cereus* strains with catalase explicitly abolished the growth-inhibitory activity of all three types of berries. Lower concentrations, up to 1000 U, were sufficient to improve the enumeration efficiency for cranberry and blackcurrant cultures (Fig. 4C-F). In contrast, a significant increase in the survival of cells exposed to chokeberry juice required more than 5000 U of the enzyme (Fig. 4A, B).

### Physicochemical characteristics of berry juices

The caption under Fig. 1 provides information about the influence of different juice supplements on the initially neutral pH of the growth medium. In this regard, cranberry and blackcurrant juices exhibited much more pronounced acidification effects compared with chokeberry and elderberry juices, which was attributed to their significantly lower pH and higher TA, as indicated in Table 1.

**Table 1:** Physicochemical characteristics of the analyzed berry juices (mean ± SD).

Parameter	Chokeberry	Blackcurrant	Cranberry	Elderberry
pH	3.29 ± 0.01 ^b^	2.88 ± 0.03 ^c^	2.42 ± 0.01 ^d^	3.83 ± 0.03 ^a^
TA [mL 0.1 M NaOH/mL]	1.78 ± 0.03 ^d^	3.80 ± 0.00 ^a^	3.17 ± 0.06 ^b^	2.02 ± 0.03 ^c^
DPPH [mg TE/L]	4949 ± 9 ^a^	4019 ± 148 ^b^	936 ± 134 ^d^	2587 ± 54 ^c^
ABTS [mg TE/L]	9378 ± 8 ^a^	6506 ± 268 ^b^	2261 ± 142 ^d^	5944 ± 148 ^c^
TPc_F−C_ [mg GAE/L]	13,019 ± 281 ^a^	6558 ± 36 ^b^	2615 ± 24 ^d^	5635 ± 429 ^c^
TACNs [mg/100 mL]	9.32 ± 0.05 ^b^	1.22 ± 0.01 ^c^	0.66 ± 0.00 ^c^	239.20 ± 1.24 ^a^
TPc_UHPLC_ [mg/100 mL]	111.94 ± 0.58 ^b^	51.81 ± 0.26 ^c^	16.88 ± 0.08 ^d^	352.07 ± 1.82 ^a^

Values marked by different letters (a–d) on the same line differ significantly: α = 0.05; TA - titrable acidity; DPPH – DPPH antiradical activity; *ABTS* ABTS antiradical activity, *TPc*_***F−C***_ total phenolic content via spectrophotometry, *TACNs* total anthocyanins via UHPLC, *TPc*_UHPLC_ total phenolic content via UHPLC.

**Table 2:** Correlation coefficients between the antioxidant, acidic, and phenolic properties of the berry juices examined.

	DPPH	ABTS	TPc_F−C_	TPc_UHPLC_	TACNs	AsA	CA	TA
DPPH	**1.00**	0.96	0.91	0.03	− 0.18	− 0.29	− 0.36	N.A.
ABTS	0.96	**1.00**	0.95	0.23	0.01	− 0.53	− 0.51	N.A.
TPc_F−C_	0.91	0.95	**1.00**	0.06	-0.17	− 0.55	− 0.72	N.A.
TPc_UHPLC_	0.03	0.23	0.06	**1.00**	N.A.	N.A.	N.A.	N.A.
TACNs	− 0.18	0.01	− 0.17	N.A.	**1.00**	N.A.	N.A.	N.A.
AsA	− 0.29	− 0.53	− 0.55	N.A.	N.A.	**1.00**	N.A.	0.99
CA	− 0.36	− 0.51	− 0.72	N.A.	N.A.	N.A.	**1.00**	0.79
TA	N.A.	N.A.	N.A.	N.A.	N.A.	0.99	0.79	**1.00**

*DPPH* DPPH antiradical activity, *ABTS* ABTS antiradical activity, *TPc*_***F−C***_ total phenolic content via spectrophotometry, *TPc*_UHPLC_ total phenolic content via UHPLC, *TACNs* total anthocyanins via UHPLC, *AsA* ascorbic acid, *CA* citric acid, *TA* titrable acidity, *N.A.* not applicable.

Correlations of 1.00 for identical parameters are in bold.

Specifically, the juices were characterized by pH values ranging from 2.4 to 3.8 (Table 1), which is characteristic of this type of fruit family^27^. The pH of the chokeberry juice was greater than 3, whereas the pH of the blackcurrant and cranberry juices was less than 3. A strong negative correlation was observed between the pH and TA of the juices (*r* = − 0.71, d.n.s.). Additionally, the TA was strongly associated with the contents of AsA and CA (*r* = 0.99 and *r* = 0.79, respectively; Table 2). Consequently, blackcurrant and cranberry juices presented significantly higher levels of these organic acids compared with chokeberry juice (Table 3). With respect to AsA and CA, no positive correlation was observed between their levels and AC as determined by the DPPH method.

(*r* = − 0.29 and *r* = − 0.36, respectively) and the ABTS method (*r* = − 0.53 and *r* = − 0.51, respectively). However,

a strong positive correlation was observed between the AC and TPc_**F−C**_ (Table 2), which aligns with the findings reported by^20^. Notably, chokeberry juice had the highest TPc_**F−C**_, whereas elderberry juice presented the highest TACNs content (Table 1). Interestingly, no association was found between AC and TACNs (Table 2). Together, these findings provide an explanation for the significantly lower AC (*P* < 0.05) of blackcurrant, cranberry, and elderberry juices than of chokeberry juice (Table 1), which is in line with the results reported by^9^. Notably, chokeberry and elderberry juices were characterized by significantly elevated levels of chlorogenic and neochlorogenic acids compared with blackcurrant and cranberry juices (Table 3).

**Table 3:** Phenolic and organic acid contents in the analyzed berry juices (mean ± SD).

Phytochemicals [mg/100 mL]	Chokeberry	Blackcurrant	Cranberry	Elderberry
Phenolic compounds				
(–)-Gallocatechin	N.D.	41.61 ± 0.21 ^a^	N.D.	4.98 ± 0.03 ^b^
Procyanidin B2	0.56 ± 0.00 ^b^	N.D.	0.61 ± 0.00 ^a^	N.D.
(–)-Epicatechin	1.49 ± 0.01 ^b^	0.43 ± 0.00 ^d^	0.82 ± 0.00 ^c^	11.72 ± 0.06 ^a^
(–)-Epigallocatechin gallate	N.D.	N.D.	N.D.	1.00 ± 0.01
Quercetin 3-*O*-rutinoside	9.20 ± 0.05 ^b^	0.33 ± 0.00 ^d^	3.25 ± 0.02 ^c^	42.10 ± 0.22 ^a^
Quercetin 3-*O*-glucoside	2.87 ± 0.01 ^b^	2.16 ± 0.01 ^d^	2.61 ± 0.01 ^c^	8.81 ± 0.05 ^a^
Luteolin 7-*O*-glucoside	N.D.	N.D.	N.D.	N.D.
Quercetin 3-*O*-rhamnoside	N.D.	0.43 ± 0.00 ^b^	1.47 ± 0.01 ^a^	N.D.
Quercetin	0.29 ± 0.00 ^d^	0.47 ± 0.00 ^c^	2.19 ± 0.01 ^a^	1.77 ± 0.01 ^b^
Apigenin	N.D.	N.D.	N.D.	3.06 ± 0.02
Gallic acid	0.15 ± 0.00 ^c^	0.19 ± 0.00 ^b^	0.08 ± 0.00 ^d^	1.93 ± 0.01 ^a^
Protocatechuic acid	3.57 ± 0.02 ^b^	N.D.	1.03 ± 0.01 ^c^	4.61 ± 0.02 ^a^
4-hydroxybenzoic acid	N.D.	1.27 ± 0.01 ^a^	N.D. ^c^	1.15 ± 0.01 ^b^
Vanillic acid	N.D.	N.D.	N.D.	N.D.
Syringic acid	N.D.	N.D.	N.D.	N.D.
Ellagic acid	N.D.	N.D.	N.D.	0.84 ± 0.00
Caffeic acid	N.D.	N.D.	N.D.	2.33 ± 0.01
Neochlorogenic acid	45.66 ± 0.24 ^a^	0.71 ± 0.00 ^c^	N.D.	14.21 ± 0.07 ^b^
Chlorogenic acid	38.83 ± 0.20 ^a^	1.88 ± 0.01 ^d^	3.05 ± 0.02 ^c^	14.37 ± 0.07 ^b^
Ferulic acid	N.D.	0.35 ± 0.00 ^a^	0.13 ± 0.00 ^b^	N.D.
*p*-Coumaric acid	N.D.	0.76 ± 0.00 ^b^	0.99 ± 0.01 ^a^	N.D.
Organic acids				
Ascorbic acid	64.60 ± 0.70 ^c^	263.20 ± 13.90 ^a^	216.40 ± 3.10 ^b^	65.40 ± 2.80 ^c^
Citric acid	11.20 ± 0.17 ^d^	2,529.60 ± 92.50 ^a^	1,544.40 ± 20.90 ^c^	1,702.90 ± 58.30 ^b^
Tartaric acid	94.70 ± 6.50 ^b^	N.D.	264.00 ± 3.80 ^a^	65.60 ± 5.40 ^c^
Oxalic acid	22.60 ± 2.30 ^c^	51.70 ± 1.40 ^b^	N.D.	274.90 ± 5.10 ^a^
Fumaric acid	0.20 ± 0.00 ^c^	1.70 ± 0.20 ^b^	N.D.	4.40 ± 0.10 ^a^
Malic acid	1,197.90 ± 15.00 ^a^	318.70 ± 1.10 ^c^	558.50 ± 8.10 ^b^	153.80 ± 12.70 ^d^

Values marked by different letters (a–d) on the same line differ significantly: α = 0.05.

*N.D.* not detected.

## Discussion

An alternative strategy proposed for mitigating *B. cereus* contamination in minimally processed foods involves targeting vegetative cells^4^. Given the potential for initial spores to germinate, applying (bio)preservatives to deactivate or control the growth of subsequent vegetative cells can effectively reduce the prevalence of this bacterium. In this study, we investigated the biopreservation potential of juices derived from phenolic-rich elderberry and chokeberry fruits against *B. cereus* vegetative cells while also examining the effects of.

vitamin C-rich blackcurrant and cranberry juices for comparison.

Particular attention should be given first to antibacterial evaluation involving catalase (Fig. 4). The substantial colony-forming capacity of enzyme-treated *B. cereus* cultured under predefined growth-inhibitory conditions indicates that the cell death induced by the examined juices involves the role of hydrogen peroxide. Certainly, one could consider it an extracellular byproduct resulting from the autooxidation processes of polyphenols and/or organic acids derived from juices (Table 3)^28^. Indeed, the accumulation of H_2_O_2_ in commonly exploited cell culture media upon supplementation with specific antioxidant substances has been well documented^29^. However, it is crucial to consider the elevated rate of autooxidation processes with increasing pH, particularly concerning ascorbic acid^28,29^. Given the absence of inhibitory effects observed for neutralized vitamin C-rich blackcurrant and cranberry supplements, the concept of extracellular H_2_O_2_-mediated toxicity of the juices should be dismissed. In contrast, the findings of^30–33^ suggest that the antibacterial activity of juices could operate directly at the intracellular level. These studies reported increased formation of reactive oxygen species (ROS), including hydrogen peroxide, as well as the induction of catalase-encoding genes in *B. cereus* cells when directly exposed to compounds of either phenolic (epigallocatechin gallate) or organic acid (lactic and acetic acids) nature. Therefore, it is conceivable that certain juice components could intercalate or permeate the phospholipid membrane of *B. cereus* cells, thereby disrupting the aerobic electron transport chain and consequently resulting in cellular catalase insufficiency, hydrogen peroxide accumulation, oxidative damage, and ultimately, cell death. Interestingly, the most potent chokeberry and blackcurrant juices (Fig. 1) presented the highest DPPH antiradical activity (Table 1), suggesting that hydrophobic antioxidants^34^ acted as pro-oxidative.

growth-inhibitory agents when entering bacterial cells at sufficiently high concentrations. Given the determining influence of extracellular pH on the acid‒base behavior of individual juice constituents and thus their interactions with negatively charged membranes of *B. cereus* cells, neutralization assessment may provide valuable insights into the chemical nature of the antibacterials present in juices.

Importantly, fully protonated organic acids are known to dominate only at pH values below 5^35^. In contrast, certain polyphenols, such as cyanidins, may exist predominantly as uncharged species even at pH values ranging from 6.39 to 6.9, as supported by data from^36^ and the FooDB database based on ChemAxon software. Therefore, the strict pH dependence observed in the antibacterial effectiveness of blackcurrant and cranberry juice supplements (Fig. 1 C-F) suggests that their antagonism was likely due to organic acids, whereas for chokeberry juice, it is believed to have primarily stemmed from phenolics, conceivably anthocyanins. Indeed, this particular fraction of *Aronia melanocarpa* juice, containing cyanidin-3-*O*-galactoside as the predominant compound, notably reduced the viability of *B. cereus* after 24 h of treatment, as indicated in Fig. 3A. On the other hand, the elevated content of AsA and CA in blackcurrant juice (Table 3), along with its inherently increased acidity (Table 1), likely contributed to its superior inhibitory effectiveness in relation to that of cranberry juice (Fig. 1). Previous studies highlighting the significant discrepancy in antibacterial efficacy between berry-derived organic acids and phenolics under neutral pH conditions provide additional support for the drawn conclusions^9,37–40^.

In these contexts, observing the “growth phenotype” of *B. cereus* when subjected to elderberry juice, which is reported to be exceptionally rich in anthocyanins, is particularly intriguing (Table 1). Similarly, the difference in antibacterial capacity between neutralized blackcurrant juice (Fig. 2C-D) and its anthocyanin fraction (Fig. 3C) requires further elucidation. On the basis of the proposed mechanisms of susceptibility in *B. cereus*, it is speculated that the anthocyanins included within elderberry and blackcurrant juice supplements lacked the physicochemical capacity to effectively penetrate the bacterial cell barrier. Considering the partition coefficient (logP) of a compound as a measure of its lipophilicity^40^, it is tempting to examine this aspect in relation to the predominant anthocyanins found in the examined juices: cyanidin-3-*O*-galactoside (chokeberry juice),

cyanidin-3-*O*-rutinoside (blackcurrant juice), and cyanidin-3-*O*-sambubioside (elderberry juice). It is reasonable to assume that a higher partition coefficient may result in a more favorable interaction with the bacterial membrane, leading to greater inhibitory effectiveness. This concept appears to apply particularly to phenolic compounds rather than organic acids^40^. Notably, the predicted logP values for the aforementioned anthocyanins are approximately 0.39, -0.64, and − 1.1, respectively, according to the FooDB database based on ChemAxon software. In this context, the results presented by^17^, who reported the inferior lipophilic antioxidant capacity (L-ORAC_FL_) of elderberry and blackcurrant fruits relative to that of chokeberry, provide further support for the suggested concept of *B. cereus* antibacterial susceptibility to the examined juices and their fractional components (Figs. 1 and 3). Second, the potent antibacterial activity of the anthocyanins derived from blackcurrant juice (Fig. 3C), even when they are applied at concentrations as low as approximately 0.3 µg/mL (d.n.s.), suggests that specific constituents of this juice may limit the effectiveness of cyanidin-3-*O*-rutinoside and/or other phenolics when used in combination under neutral pH conditions. This finding highlights the potential complexity of interactions between anthocyanins and bacterial pathogens with respect to specific whole juice supplements. Further investigations are warranted to elucidate the role of ascorbic acid copresence^41^ in this context.

## Conclusions

Our study aimed to elucidate the antibacterial efficacy of selected berry juices against *B. cereus*. The juices originate from four different berry species, namely, *Aronia melanocarpa*, *Ribes nigrum*, *Vaccinium macrocarpon*, and *Sambucus nigra*. We conducted a comparative analysis of the phenolic and organic acid compositions of the juices, as well as their physicochemical properties, such as acidity and antioxidant capacity. Antibacterial assessments are performed on both laboratory and environmental strains of *B. cereus*, with a focus on native and neutralized juices, as well as their fractional components, including anthocyanins. Additionally, we introduce the catalase enzyme to provide further insights into our investigation.

It is inferred that the antibacterial susceptibility of *B. cereus* to juices is associated with pro-oxidative effects induced directly at the intracellular level. This highlight a strict correlation between the growth-inhibitory activity of specific berry-derived components and their antioxidant capacity, as well as their intrinsic pKa (acidity) and/or logP (lipophilicity) values. As such, organic and phenolic acids are considered most effective under low extracellular pH conditions, whereas lipophilicity is crucial for the activity of anthocyanins.

In summary, elderberry juice lacks antibacterial activity due to its low total acidity and possibly poor lipophilic antioxidant capacity, despite being rich in anthocyanins, chlorogenic acid, neochlorogenic acid, and citric acid. Consequently, the introduction of natural sugars within the supplements likely stimulates bacterial metabolism and promotes cellular growth. Hence, elderberry juice is not recommended for biopreservative applications against *B. cereus*. On the other hand, significant acidification not only results from the use of organic acid-rich and phenolic-deficient blackcurrant and cranberry juices but is also crucial for their antibacterial effectiveness, which is considered a limitation from a biopreservation perspective. Finally, chokeberry juice has emerged as the most potent inhibitor of *B. cereus* growth conceivably due to the exceptionally high antioxidant capacity and seemingly strong lipophilic properties of the anthocyanins present. Overall, this study contributes to our understanding of the antibacterial susceptibility of *B. cereus*.

## Electronic supplementary material

Below is the link to the electronic supplementary material.

Supplementary Material 1

## Data Availability

The datasets used and/or analyzed during the current study are available from the corresponding author on reasonable request.
