# Supplementary material for: Antibacterial efficacy of berry juices against Bacillus cereus relative to their phytochemical composition and antioxidant properties
Source: Sci Rep. 2024 Nov 16;14:28298. doi: 10.1038/s41598-024-79155-y (PMC11569148; doi:10.1038/s41598-024-79155-y)
Supplement: Supplementary file 1 — Supplementary Material 1 [file 41598_2024_79155_MOESM1_ESM.docx]

***Supplementary material***

**Antibacterial efficacy of berry juices against *Bacillus cereus* relative to their phytochemical composition and antioxidant properties**

**Kamil Królak^1^*** ∙ **Sylwia Ścieszka^1^** ∙ **Edyta Kordialik-Bogacka^1^** ∙ **Joanna Oracz^2^** ∙ **Maciej Ditrych^1^** ∙ **Tomasz Szczygieł^1^** ∙ **Katarzyna Dybka-Stępień^1^** ∙ **Anna Otlewska^1^** ∙ **Dorota Żyżelewicz^2^**

1. Institute of Fermentation Technology and Microbiology, Faculty of Biotechnology and Food Sciences, Lodz University of Technology, Lodz, Poland; [kamil.krolak@dokt.p.lodz.pl](mailto:kamil.krolak@dokt.p.lodz.pl) (K.K.); [sylwia.scieszka@p.lodz.pl](mailto:sylwia.scieszka@p.lodz.pl) (S.Ś.); [edyta.kordialik-bogacka@p.lodz.pl](mailto:edyta.kordialik-bogacka@p.lodz.pl) (E.K-B.); [maciej.ditrych@p.lodz.pl](mailto:maciej.ditrych@p.lodz.pl) (M.D.); [tomasz.szczygiel@dokt.p.lodz.pl](mailto:tomasz.szczygiel@dokt.p.lodz.pl) (T.S.); [katarzyna.dybka@p.lodz.pl](mailto:katarzyna.dybka@p.lodz.pl) (K.D.-S.); [anna.otlewska@p.lodz.pl](mailto:anna.otlewska@p.lodz.pl) (A.O.);
2. Institute of Food Technology and Analysis, Faculty of Biotechnology and Food Sciences, Lodz University of Technology, Lodz, Poland; [joanna.oracz@p.lodz.pl](mailto:Joanna.oracz@p.lodz.pl) (J.O.); [dorota.zyzelewicz@p.lodz.pl](mailto:dorota.zyzelewicz@p.lodz.pl) (D.Ż.).

*Corresponding author: Kamil Królak, [kamil.krolak@dokt.p.lodz.pl](mailto:kamil.krolak@dokt.p.lodz.pl).

**Table S1.** The range of calibration, the coefficient of determination (R^2^), limit of detection (LOD), and limit of quantification (LOQ) of the investigated phenolic compounds determined by UHPLC-DAD.

| Standard | Linear range  (mg/L) | R2 | LOD (mg/L) | LOQ (mg/L) |
| --- | --- | --- | --- | --- |
| (–)-Gallocatechin | 0.1-50.0 | 0.995 | 0.07 | 0.20 |
| (–)-Epicatechin, | 0.1-50.0 | 0.997 | 0.04 | 0.12 |
| (–)-Epigallocatechin gallate, | 0.1-50.0 | 0.996 | 0.05 | 0.14 |
| Procyanidin B2 | 0.1-50.0 | 0.999 | 0.04 | 0.12 |
| Procyanidin C1 | 0.1-50.0 | 0.997 | 0.04 | 0.12 |
| Quercetin 3-*O*-rutinoside | 0.1-50.0 | 0.997 | 0.02 | 0.08 |
| Quercetin 3-*O*-glucoside | 0.1-50.0 | 0.999 | 0.02 | 0.08 |
| Quercetin 3-*O*-rhamnoside | 0.1-50.0 | 0.995 | 0.02 | 0.08 |
| Luteolin 7-*O-*glucoside | 0.1-50.0 | 0.998 | 0.02 | 0.08 |
| Quercetin | 0.1-50.0 | 0.998 | 0.03 | 0.10 |
| Apigenin | 0.1-50.0 | 0.995 | 0.03 | 0.10 |
| Gallic acid | 0.1-50.0 | 0.999 | 0.01 | 0.03 |
| 4-Hydroxybenzoic acid | 0.5-100.0 | 0.995 | 0.04 | 0.10 |
| Protocatechuic acid | 0.5-100.0 | 0.997 | 0.03 | 0.09 |
| Ellagic acid | 0.5-100.0 | 0.999 | 0.08 | 0.24 |
| Caffeic acid | 0.1-50.0 | 0.998 | 0.04 | 0.12 |
| Neochlorogenic acid, | 0.1-50.0 | 0.996 | 0.03 | 0.10 |
| Chlorogenic acid | 0.1-50.0 | 0.999 | 0.03 | 0.10 |
| *p*-Coumaric acid | 0.1-50.0 | 0.997 | 0.03 | 0.09 |
| Ferulic acid | 0.1-50.0 | 0.995 | 0.03 | 0.09 |
